# Effect of pelvimetric diameters on success of surgery in patients submitted to robot-assisted perineal radical prostatectomy

**DOI:** 10.1590/S1677-5538.IBJU.2019.0413

**Published:** 2020-02-20

**Authors:** Mustafa Gurkan Yenice, Ismail Yigitbasi, Rustu Turkay, Selcuk Sahin, Volkan Tugcu

**Affiliations:** 1 University of Health Sciences Istanbul Bakirkoy Dr. Sadi Konuk Training and Research Hospital Department of Urology Istanbul Turkey Department of Urology, University of Health Sciences, Istanbul Bakirkoy Dr. Sadi Konuk Training and Research Hospital, , Istanbul, Turkey; 2 University of Health Sciences Istanbul Bakirkoy Dr. Sadi Konuk Training and Research Hospital Department of Radiology Istanbul Turkey Department of Radiology, University of Health Sciences, Istanbul Bakirkoy Dr. Sadi Konuk Training and Research Hospital, Istanbul, Turkey

**Keywords:** Prostatic Neoplasms, Prostatectomy, Perineum

## Abstract

**Objective:**

Minimally invasive techniques are used increasingly by virtue of advancements in technology. Surgery for prostate cancer, which has high morbidity, is performed with an increasing momentum based on the successful oncological and functional outcomes as well as cosmetic aspects.

**Materials and methods:**

Sixty two patients underwent robot-assisted perineal radical prostatectomy (R-PRP) surgery at our clinic between November 2016 and August 2017. Six pelvimetric dimensions were defined and measured by performing multiparametric magnetic resonance imaging (mpMRI) prior to operation in all patients. In light of these data, we aimed to investigate the effect of pelvimetric measurements on surgery duration and surgical margin positivity.

**Results:**

By using this technique in pelvic area, we observed that measurements only representing surgical site and excluding other pelvic organs had a significant effect on surgery duration, and pelvic dimensions had no significant effect on surgical margin positivity.

**Conclusion:**

In R-PRP technique, peroperative findings and oncological outcomes can vary depending on several variable factors, but although usually not taken into account, pelvimetric measurements can also affect these outcomes. However, there is a need for randomised controlled trials to be conducted with more patients.

## INTRODUCTION

As the prostate anatomy and distribution of neurovascular bundle (NVB) have been better understood, radical prostatectomy has become increasingly used as part of multimodal treatment to eradicate the disease in local-stage cancer and high-risk prostate cancer (1). While radical prostatectomy results in eradication of disease, open, laparoscopic or robot-assisted radical prostatectomy (RALP) can be performed by considering benefits and side effects of the technique (2). According to literature findings, pelvic dimensions were determined to be an important impact factor in oncological and functional outcomes of surgical methods used. Apical prostate depth is an important impact factor for laparoscopic radical prostatectomy (LRP) and retropubic radical prostatectomy (RRP) (3). Another important study showed that the pelvic diameter measurements were not predictive of improvement in erectile function in patients underwent RRP (4). Based on the evidence from studies of RALP method, pelvimetric dimensions were proved to be ineffective for surgery duration, amount of bleeding, and recovery of potency and continence at 6-month follow-up of patients underwent this technique (5).

Unlike conventional methods, robot-assisted perineal radical prostatectomy (R-PRP) is performed through perineal approach. In addition, it has many advantages in patients with a history of major abdominal surgery and high body mass index. As this technique is performed below the endopelvic fascia and bladder neck level, anatomy and physiology created by several prior surgeries such as kidney transplantation or intestinal bypass systems for colorectal cancer can be maintained. Tugcu et al. performed this technique in 15 patients and reported that it can be safely performed in terms of oncological and functional outcomes (6). In this study, we aimed to investigate the effect of pelvimetric diameters measured preoperatively by multiparametric magnetic resonance imaging on peroperative findings and postoperative oncological outcomes.

## MATERIALS AND METHODS

Based on our database collected prospectively, 62 patients received R-PRP treatment for prostate cancer between November 2016 and August 2017. All mpMRI images were obtained using 3 Tesla MRI machine (Magnetom Verio; Siemens, Erlanger, Germany). Sequences taken were T1--weighted axial and T2-weighted triplanar (axial, sagittal and coronal) and diffusion-weighted images (b values were calculated to be 0, 400, 800, 1400 and 1400). mpMRI did not reveal extraprostatic spread in any of the patients. We performed R-PRP method by placing a gel-port platform on the potential space, which was demarcated by rectourethral muscles and created by open perineal dissection, followed by robotic procedure. All the cases were performed by a single surgeon who has advanced experiences on robotic surgery. In our study, 6 pelvimetric dimensions were measured by an experienced radiologist. The first dimension consists narrowest distances between tips of the ischial spines (ISD) in T2-weighted axial images (Figure-1a). Unlike other techniques in R-PRP, surgery is performed in only minor pelvis with perineal approach. The second dimension is composed angle of the intersection of the straight lines extending from the tuber ischiadicum to the symphysis pubis (ASP) in the T2 weighted sequence coronal images (Figure-1b). We think that this angle represents the minor pelvis. The third dimension is the anteroposterior diameter of pelvic midplane between lower tip of the symphysis pubis and the coccyx representing the pelvic outlet (DPO) (Figure-1c). In other techniques, pelvic inlet and outlet are important because of the operation with abdominal approach, in R-PRP only pelvic outlet is important because technique is performed below endopelvic fascia level. The fourth dimension is the distance of the pelvic midplane from the anterior border of the anus to the apex of the prostate in sagittal images (DAA) (Figure-2a). The fifth dimension is the distance of the pelvic midplane from the anterior border of the anus to anterior border of the centre of seminal vesicles in sagittal images (DSA) (Figure-2b). The sixth dimension is the angle formed by the intersection of the axis passing through the lower and upper tips of the symphysis pubis and the axis intersecting of the seminal vesicles in cranio-caudal line on the sagittal midplane of pelvis (ASS) (Figure-2c). This angle represents the area below the level of endopelvic fascia when the rectum is excluded from the minor pelvis. In addition, prostate volume and body mass index of patients were measured and the effects of results on open perineal procedure time, console time and total operative time, and perioperative findings and postoperative oncological outcomes were examined.

**Figure 1A:**
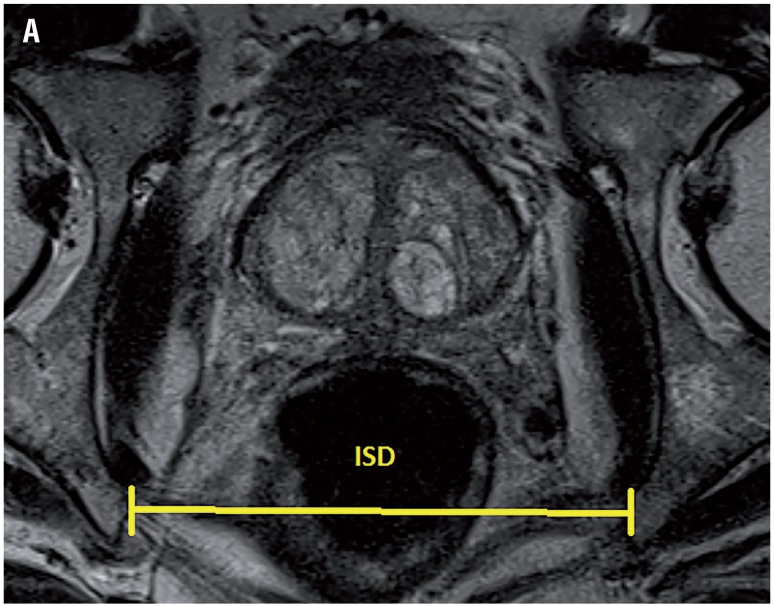
Distance of the ischial spines.

**Figure 1B:**
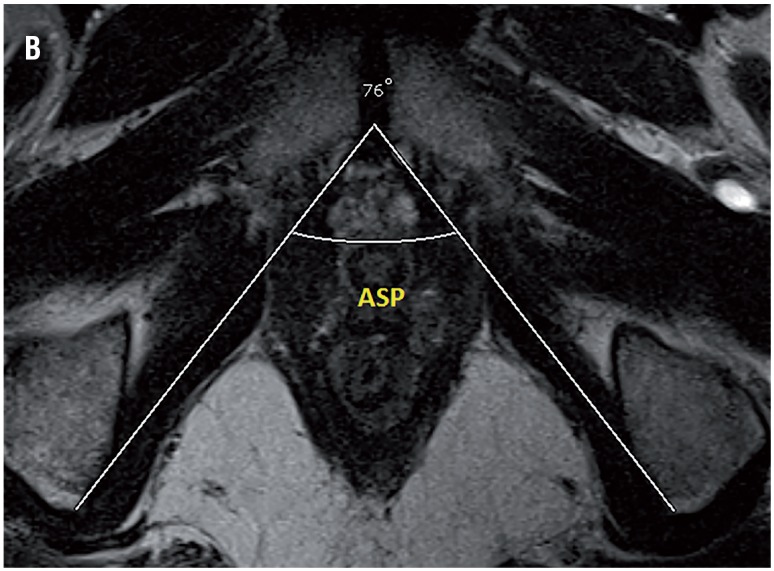
Angle of symphysis pubis.

**Figure 1C:**
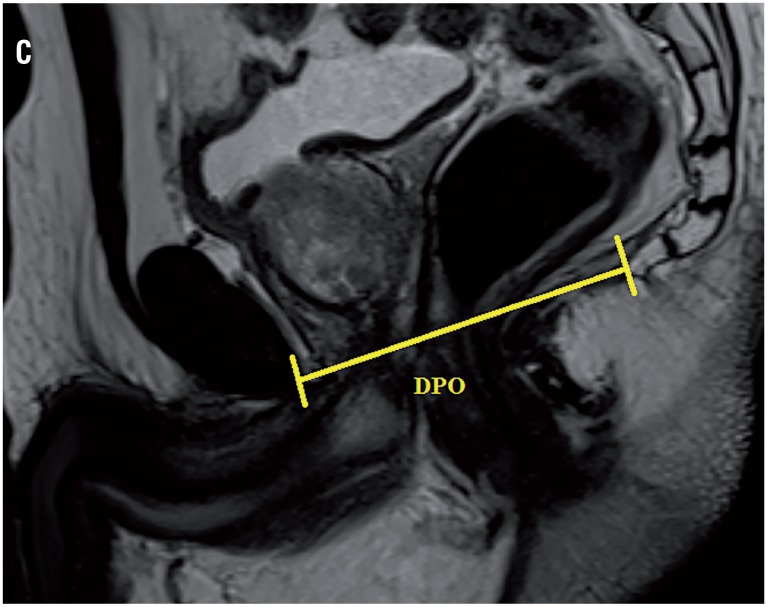
Distance of pelvic outlet.

**Figure 2a:**
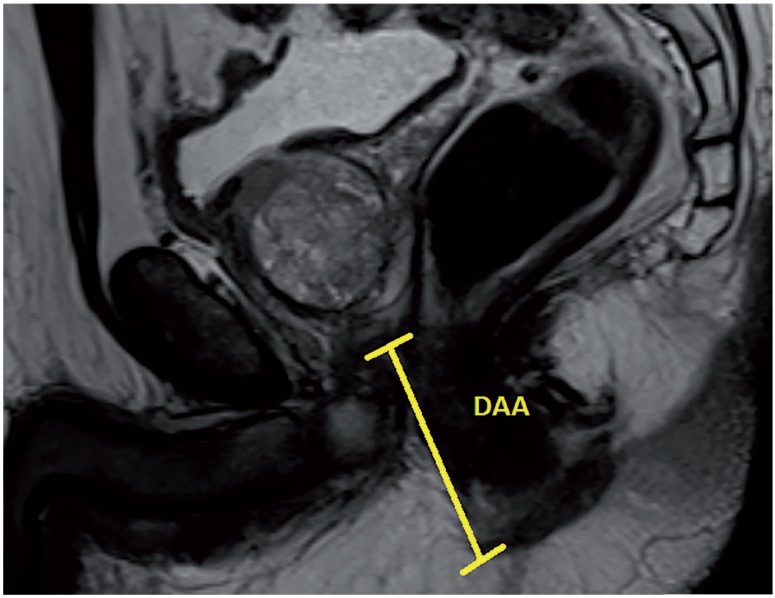
Distance between prostate apex and anus.

**Figure 2b:**
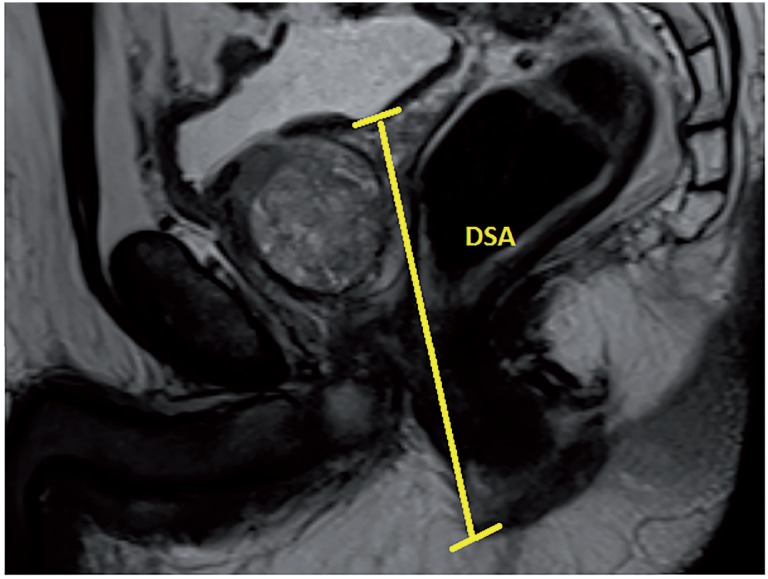
Distance between seminal vesicles and anus.

**Figure 2C:**
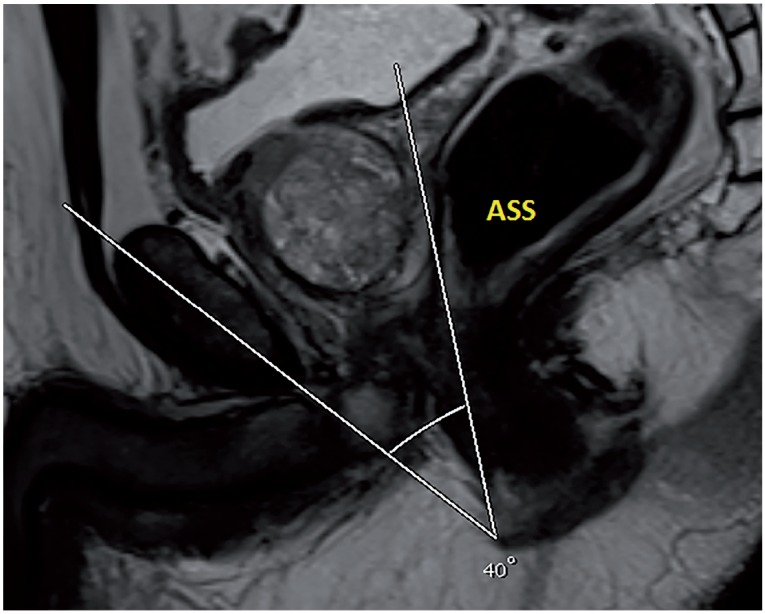
Angle of symphysis pubis-seminal vesicles.

### Statistical analysis

Descriptive statistics were used to define continuous variables (mean, standard deviation, minimum, median, maximum). Multiple linear regression analysis was performed to examine the effect of independent variables on continuous dependent variables. Statistical significance level was considered to be 0.05. Analyses were performed using MedCalc Statistical Software version 12.7.7 (MedCalc Software bvba, Ostend, Belgium; 2013).

### Surgical technique

In the first stage, surgery was initiated by performing an open perineal dissection. A potential space was created by extending the dissection into the rectourethral muscle fibres. GelPOINT^®^ (Applied Medical, Rancho Santa Margarita, CA, USA) was placed on this space with the patient on 15-degree Trendelenburg and exaggerated lithotomy position. Three 8mm robotic trocars and one 12mm assistant trocar were placed on gel port. Trocar on 7 o'clock position was used for bipolar robotic arm and robotic trocar on 5 o'clock position was used for scissors and large needle robotic arm. Assistant trocar was placed on 6 o'clock position (Figure-3a). Robot was docked and robot-assisted perineal radical prostatectomy was performed using 3-arm Da Vinci Xi HD Surgical System (Intuitive Surgical, Inc., Sunnyvale, CA, USA). Prostate was released by dissecting lateral prostate lobes starting from apex dissection. Seminal vesicles were completely dissected. Urethra was cut following the dissection starting from dorsal plane. Urethral catheter removed from urethra was clipped with a Hem-o-Lock clip. Urethral catheter to be used for traction was cut below the clips by maintaining the insufflation of balloon. Dorsal veins were released by venous-preserving dissection. Prostatic pedicles were dissected and Hem-o-Lock^®^ Clip (Teleflex Medical, Research Triangle Park, North Carolina, USA) was placed and cut (Figure-3b). Bladder neck was dissected and cut (Figure-3c). After completion of prostatectomy, vesicourethral anastomosis was performed by modified Van Veltoven technique using 2/4/V-Loc™ (Covidien, Mansfield, MA, ABD) sutures (Figure-3d).

**Figure 3A:**
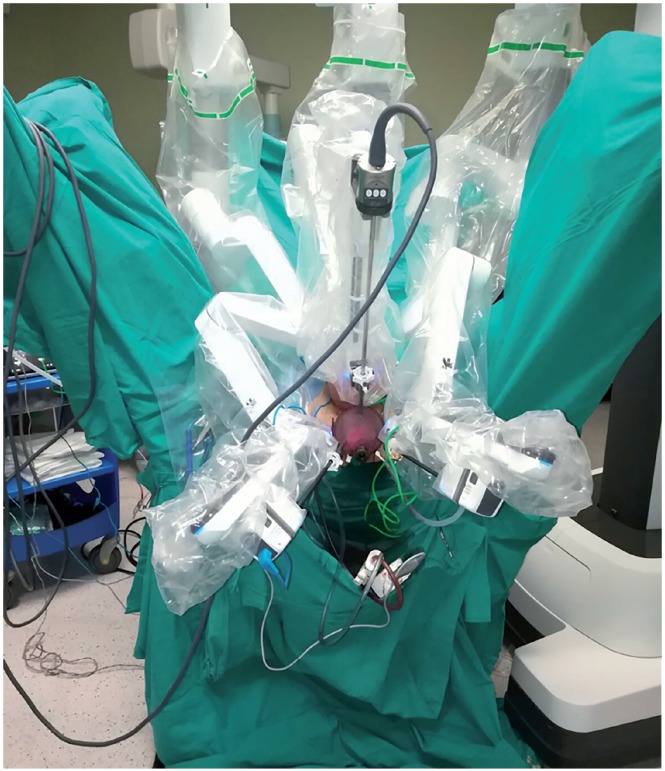
Trocar placement and docking for R-PRP.

**Figure 3B:**
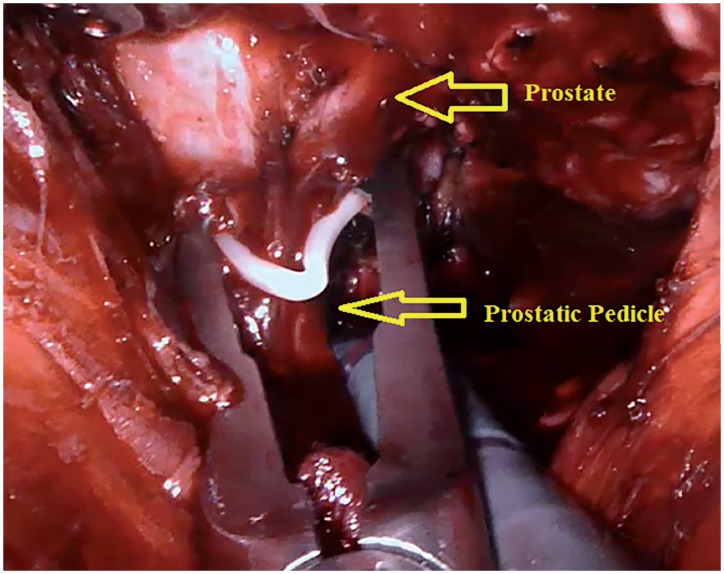
Dissection of prostatic pedicles.

**Figure 3C:**
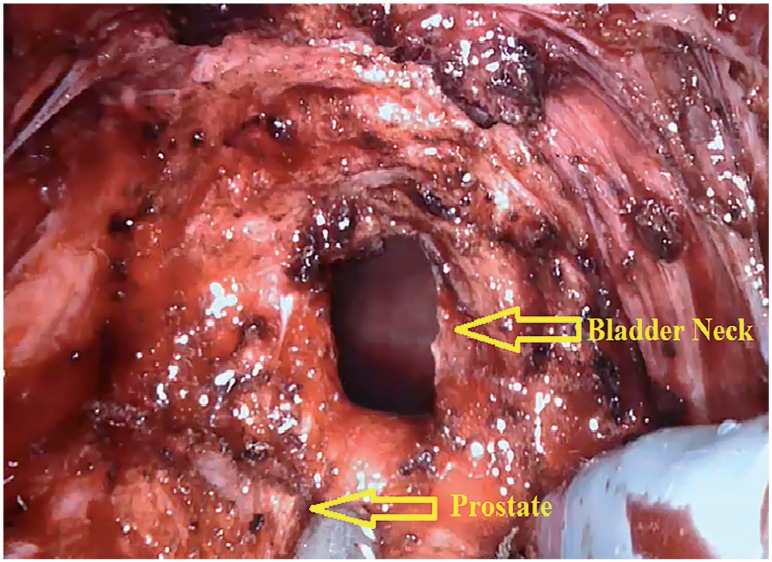
Dissection of bladder neck.

**Figure 3D:**
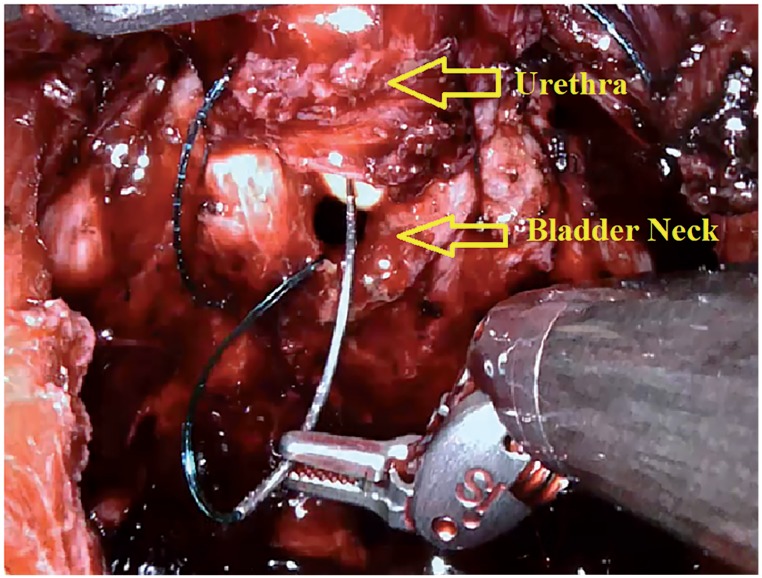
Vesico-urethral anastomosis in R-PRP.

## RESULTS

Patient's preoperative demographics, peroperative and postoperative data and pelvimetric measurement results are summarised in Tables 1, 2, 3 and 4, respectively. Results show that the open perineal dissection time is affected by ASS and DSA dimensions during the first phase of surgery. An inverse proportion was observed with ASS (p=0.004), while a direct proportion was seen with DSA (p=0.013). Open perineal dissection time was increased 0.242-fold with a 1-degree decrease in ASS and 0.277-fold with a 1mm increase in DSA. No significant statistical relationship was observed between other measurements and open perineal dissection time. During the second phase of surgery, console time was observed to be affected inversely proportional to ASP (p=0.024) and ASS (p <0.001) dimensions. Console time was increased 1.040-fold with a 1-degree decrease in ASP and 0.845-fold with a 1-degree decrease in ASS. Total operative time was affected inversely proportional to ASP (p=0.031) and ASS (p <0.001) dimensions. It was increased 1.030-fold with a 1-degree decrease in ASP and 0.875-fold with a 1-degree decrease in ASS. Prostate volume did not cause a change in open perineal dissection time. Body mass index and prostate volume were not observed to have a statistical effect on console time and total operative time. Open perineal dissection time was found to be inversely proportional to body mass index of patient (p=0.038) and increased 0.974-fold with each increment in body mass index. Blood loss was observed to be affected inversely proportional to ASP (p=0.024) and ASS (p=0.003), and directly proportional to DSA (p=0.023). Blood loss was increased 1.456-fold with a 1-degree decrease in ASP, 1.758-fold with a 1-degree decrease in ASS and 1.160-fold with a 1-degree increase in DSA (Table-5).

**Table 1:** Demographic and the preoperative data.

		r-PRP
Mean age (Range)		63.4 (46-73)
Mean BMI (kg/m^2^-Range)		28.5 (24-32)
Mean PSA (ng/mL-Range)		7.35 (3.92-17.3)
Prostate Volume (cc-Range)		69.8 (25-140)
**Previous abdominal/Pelvic Surgery**		
	Yes	40 (64%)
	No	22 (36%)
**ASA score**		
	1	5 (4%)
	2	54 (87%)
	3	5 (8%)
**Charlson score**		
	≤2	58 (89%)
	>2	4 (11%)
**Clinical stage**		
	T1c	6 (9%)
	T2a	10 (16%)
	T2b	18 (29%)
	T2c	28 (45%)
**Gleason Score**		
	6	44 (71%)
	3+4	18 (29%)

**Table 2:** Peroperative data.

	r-PRP
Mean console time (Minute-Range)	96.3 (55-160)
Open perineal dissection time (Minute-Range)	45.4 (30-65)
Mean blood loss (cc-range)	75.8 (40-145)
Anastomosis Time (Minute-Range)	11.8 (10-19)

**Table 3:** Postoperative Data.

Mean hospitalization (Day-range)	1.92 (1-3)
Mean bladder catheterization time (Day-range)	8.54 (5-12)
Positive surgical margin	(8%)
T2a	0
T2b	2 (3.2%)
T2c	3 (4.8%)

**Table 4:** Pelvimetric Dimensions.

Mean distance of the ischial spines (ISD) (mm)	75.1 (49.2-107.1)
Mean angle of symphysis pubis (ASP) (degree)	67.3 (55-77)
Mean distance of pelvic outlet (DPO) (mm)	92.4 (86-112)
Mean distance between prostate apex and anus (DAA) (mm)	52.1 (32.1-70.1)
Mean distance between seminal vesicles and anus (DSA) (mm)	74.1 (55.7-93)
Mean angle of symphysis pubis-seminal vesicles (ASS) (degree)	59.02 (34-86)

**Table 5:** Effects of Pelvimetric Dimensions.

AOT	CT	OPDT	EBL	CMP	p value
**ISD**	0.946	0.806	0.145	0.746	0.148
**ASP**	0.031	0.024	0.230	0.024	0.412
**DPO**	0.868	0.930	0.640	0.678	0.618
**DAA**	0.765	0.912	0.162	0.246	0.112
**DSA**	0.781	0.548	0.013	0.023	0.092
**ASS**	<0.001	<0.001	0.004	0.003	0.326
**PV**	0.428	0.821	0.128	0.164	0.228
**BMI**	0.816	0.732	0.038	0.326	0.712

**ISD** = Distance of the ischial spines; **ASP** = Angle of symphysis pubis; **DPO** = Distance of pelvic outlet; **DAA** = Distance between prostate apex and anus; **DSA** = Distance between seminal vesicles and anus; **ASS** = Angle of symphysis pubis-seminal vesicles; **PV** = Prostate volume; **BMI** = Body mass index; **AOT** = All operation time; **CT** = Console time; **OPDT** = Open perineal dissection time; **EBL** = Estimated blood loss; **CMS** = Surgical margin status.

## DISCUSSION

Recently defined, R-PRP technique is performed by a different anatomic approach and unlike conventional radical prostatectomy techniques, it causes little to no damage to physiological system created by prior intraabdominal surgeries. It may overcome many difficulties and provides additional advantages in cases where surgical treatment is usually not preferred as a treatment option due to the additional morbidity caused by conventional methods. For example, anatomic problems, intestinal injury risk and dissection of intestinal adhesions to reach the prostate area are the main challenges faced by surgeons in patients with prior history of abdominal surgery. Therefore, serious negative effects can be observed in postoperative oncological and functional outcomes. This technique is also compliant with anatomy and physiology created by prior surgeries. For example, in patients with a history of surgery such as kidney transplantation, prostate-sparing radical cystectomy and orthotopic urinary diversion and intestinal bypass for malignancy, it maintains the physiology of these surgeries postoperatively. Many surgeons try to maintain the oncological and functional outcomes despite the additional morbidities caused by prior surgeries. Thus, R-PRP provides a great convenience for surgeons in these cases and helps them cope with such issues.

Unlike other methods, this technique is performed with an instrument manoeuvre capability of 540 degrees under the high-resolution image of robotic system at a different compartment in a narrow area. However, there may be some factors that can be considered as limitations of this technique such as different pelvimetric measurements obtained in individual patients. This technique combines open and robotic surgeries and thus, requires a good knowledge on the anatomy of lower abdomen and pelvic floor. Especially during the open perineal dissection phase, rectum and surrounding soft tissue injuries may occur and lead to a process that can cause mortality. Rectum is not completely dissected during open perineal dissection phase, but mostly dissected and completely separated from prostate during robotic surgery phase. Korman et al. reported a rectal injury rate of 1-11% in a series of open perineal radical prostatectomy, Amorim et al. reported a rectal injury risk of 2.2-2.4% in laparoscopic extraperitoneal radical prostatectomy and Tewari et al. reported a rectal injury rate of 0.5-1.5% in a series of robotic radical prostatectomy (7–9). No rectal injury was observed in our series of R-PRP. Although it is believed that surgeons may experience some difficulties with the narrowing of pelvic area, good knowledge on pelvic and perineal anatomy, high-resolution images of robotic system and high level of experience in robotic surgery play important roles in success of this surgery.

According to study conducted by Violette PD, blood loss, preoperative PSA, robot malfunction and prostate volume are independent factors that prolong the operative time in RALP. In addition, mean operative time was reported to be 187 minutes (10). While completion of learning curve by the surgeon is another important factor, prior history of surgery and BMI are other factors that should be considered. Our study included 62 cases and we believe that this number of cases is sufficient for completion of learning curve. Mean operative time in our study can be considered acceptable when compared to other techniques. When all these data are ignored, pelvimetric dimensions can also be suggested as independent predictive factors for R-PRP. In result of their evaluation on prostate volume in terms of RALP, Hong et al. reported that the surgery duration, but not the pelvimetric dimensions, can be considered as an independent predictor (11). We believe that the pelvimetric measurement can have a high degree of significance if it covers surgical site only and excludes other organs in order to be considered as a predictive factor for the effect of pelvimetric dimensions on operative time. We assessed the operation in 3 phases individually when evaluating our technique based on the pelvimetric measurements. These were open perineal dissection, console time and total operative time. Among our pelvimetric measurements, ASS was found to be statistically significant for duration of all surgical procedures and each surgical phase became shorter individually with the increase in ASS. We believe that ASS significantly affects each phase as it only represents the surgical site where operation was performed. Our technique is performed in front of the rectum below the bladder neck and endopelvic fascia level using perineal approach. Therefore, while pelvic outlet can be a predictor, pelvic inlet cannot be a predictive factor because it is located completely outside the surgical site. ASP angle was found to be significant for total operative time and console time but with a lesser statistical significance compared to ASS since ASP angle included rectum and surrounding soft tissues. While DSA and BMI affect open perineal dissection time, these parameters do not affect other procedure times. As perineal approach was adopted, DSA particularly represents the mean length of prostate depth. This can be considered as a cause of its effect on open perineal dissection time.

Many studies revealed that the parameters such as BMI, prostate volume, intraabdominal pressure and patient's age at the time of robotic or laparoscopic radical prostatectomy are predictive factors for blood loss. Apart from these, it can be suggested that the pelvimetric measurements play a role in blood loss. Hong et al. reported that pelvimetric measurements do not affect the amount of blood loss in RALP operation but prostate volume is a predictive factor (11). According to data of our study, ASS, ASP and DSA measurements have an effect on blood loss. Surgical site becomes broader with the increase in ASS and ASP and performance of radical prostatectomy becomes easier with R-PRP technique. Bleeding can be controlled more effectively with the increased manoeuvre capability at this broadened area. With the increase in DSA, operation is performed at a deeper site in perineal area, which results in increased levels of blood loss. Based on the literature, blood loss is known to be reduced in RALP technique compared to other methods. Consistent with the literature, mean blood loss during RALP surgery was measured to be 190cc in our study (12, 13). For R-PRP technique, positive pressure on a narrow area, high-level robotic optical resolution and lack of need for dorsal vein complex dissection and ligation below the endopelvic fascia level are among the most important factors that reduce blood loss.

In the literature, Matikainen MP et al. reported that apical depth is an independent predictive factor for positive apical surgical margin (14). Tozawa et al. reported the rate of surgical margin as 26% in their series of RALP (15). Positive surgical margin rates of our series are consistent with literature. We determined that the pelvimetric diameter is not an independent predictive factor for surgical margin positivity in R-PRP technique. We believe that R-PRP can be safely performed without increasing surgical margin positivity regardless of pelvimetric measurements in localised prostate cancer.

The technique we performed is a novel and developing method. Some of the dimensions evaluated were overall pelvimetric dimensions, while others were dimensions defined by us to represent the surgical site only. We showed that some pelvimetric measurements can be considered as predictive factors for surgery duration. Number of patients can be considered as the limitation of this study.

## CONCLUSIONS

In R-PRP technique, peroperative findings and oncological outcomes can vary depending on several variable factors, but although usually not taken into account, pelvimetric measurements can also affect these outcomes. However, there is a need for randomised controlled trials to be conducted with large series of patients.

## ABBREVIATIONS

R-PRP: Robot-assisted radical perineal prostatectomy
mpMRI: Multiparametric magnetic resonance imaging
NVB: Neurovascular bundle
RALP: robot-assisted radical prostatectomy
LRP: Laparoscopic radical prostatectomy
RRP: Retropubic radical prostatectomy
ISD: Distance of the ischial spines
ASP: Angle of symphysis pubis
DPO: Distance of pelvic outlet
DAA: Distance between prostate apex and anus
DSA: Distance between seminal vesicles and anus
ASS: Angle of symphysis pubis-seminal vesicles
BMI: Body mass index
